# Fusing Wearable and Environmental Sensors for Context-Aware Firefighter Wellbeing Monitoring

**DOI:** 10.3390/s26185924

**Published:** 2026-09-19

**Authors:** Aaron Appelle, Eric Stach, Bryan Boyd, Liming Salvino, Jerome P. Lynch

**Affiliations:** Department of Civil and Environmental Engineering, Duke University, Durham, NC 27708, USA; aaron.appelle@duke.edu (A.A.); eric.stach@duke.edu (E.S.); bryan.boyd@duke.edu (B.B.); liming.salvino@duke.edu (L.S.)

**Keywords:** multimodal, firefighting, fire, wireless sensor, physiological monitoring, environmental monitoring, wellness, fatigue

## Abstract

Real-time monitoring of on-duty firefighters has the potential to reduce the risk of cardiovascular events and injuries by enabling early detection of fatigue and occupational hazards. Recent work uses wearable sensors to track metrics like heart rate and body temperature. However, most existing systems lack integrated environmental sensing, making it difficult to discern whether elevated vitals stem from physical exertion, ambient heat, or a combination of the two. To address this, a wireless sensing system is proposed that synchronizes physiological and environmental measurements during active firefighting. The system is validated using a new experiment where 28 firefighters completed repeated trials of a training circuit comprising warm-up activities and live-fire suppression. Participants answered survey questions after each phase to gauge overall wellness on a scale from 1 to 10. A predictive framework is developed to classify both the activity type and perceived wellness using the data collected. To extract useful information from the complex multimodal time histories, a series of models are compared using different sets of statistical features extracted from physiological, environmental, and demographic data. Furthermore, a linear mixed-effects (LME) model with subject-specific random intercepts is proposed to account for individual differences in biological response and self-reported wellness. Evaluation demonstrates the system differentiates warm-up from live-fire phases with over 97% accuracy. For wellness prediction, a one-label personalized LME model estimates their later ratings with a mean absolute error of 0.66±0.371, compared with 0.73±0.327 for the same model without personalization. Finally, data investigation reveals that wellness ratings were not fully explained by physiological measurements such as elevated vital signs, with individual differences and demographics accounting for most of the variance. These findings support further evaluation of multimodal sensing and personalized modeling for firefighter monitoring.

## 1. Introduction

Firefighting is known to involve physically demanding activities and occupational hazards. Firefighters must perform activities including hose operations, forced entry, ventilation, and search and rescue, all while wearing heavy personal protective equipment (PPE) [1]. These activities expose personnel to extreme heat, toxic smoke, and structural hazards. Fire suppression activities also require sustained aerobic effort. Given the combination of physical, thermal, and mental stressors associated with firefighting, the risk of cardiovascular events during and soon after duties is significantly elevated, representing the leading cause of on-the-job fatalities [1,2].

Monitoring firefighter status during active fire-suppression operations could empower fire captains and incident commanders to make timely decisions about crew rotation and rehabilitation through early identification of physiological strain and occupational hazards. Prior work has demonstrated myriad ways in which wearable physiological sensors can help monitor firefighter wellbeing. These works include wearable vests [3], heart rate and echocardiogram (ECG) sensors in straps or textiles [4,5,6,7,8], skin or body temperature sensors [9,10], and smartphone-based performance monitoring [11]. Outside of the firefighting domain, consumer wearable sensors have enabled continuous cardiovascular monitoring and personalized early warning notifications [12,13]. Other works have characterized cardiovascular responses associated with temperature, exercise, and emotions, showing the value of continuous physiological monitoring under changing conditions [14]. Given the high temperatures and toxic fumes in burning structures, other studies have focused on environmental monitoring of carbon monoxide (CO), formaldehyde, particulates, and toxic gases [15,16,17,18,19]. However, systems that integrate both physiological and environmental sensing [20,21,22,23] remain rare.

Given the persistent cardiovascular load and restrictive PPE of on-duty firefighters, real-time heart rate readings have been found to reflect an overall measure of thermal strain more than intensity of mechanical work [2,24]. Therefore, the lack of holistic multimodal sensing is a key limitation in current firefighter monitoring approaches [25]. Concurrent environmental measurements can provide context for interpreting physiological signals, including whether elevated vital signs coincide with external heat exposure. Evidence from field studies in wildfire suppression affirmed that firefighters maintained elevated heart rates and lowered heart rate variability (HRV) during rest intervals [26]. This motivates a unified multimodal sensing system that jointly characterizes environmental exposure and physiological response so that captains can make better-informed decisions about how they deploy their teams.

This work addresses the need for integrated monitoring by developing a wireless Bluetooth system that records synchronized physiological data (heart rate, body temperature, and skin temperature) and environmental conditions (ambient temperature, in-suit temperature, and humidity) during firefighting operations. The system was validated during a live-fire training experiment with 28 firefighters at Naval Submarine Base Kings Bay. The participants completed two repetitions of a strenuous training circuit consisting of scenario-based warm-up exercises carrying a fire hose, followed by live-fire suppression inside a closed CONEX container to subject the participants to high thermal load. Through a controlled experimental procedure, the research team investigated the central question of whether multimodal sensor data from the proposed system can predict firefighters’ self-reported ratings of perceived overall wellness after each phase of the experiment.

A number of challenges must be overcome to successfully predict firefighter wellbeing in this context. Firstly, it is difficult to extract predictive value from large-scale multimodal sensor data, especially in complex real-world environments. Sensor fusion approaches [27,28,29] outperform single-sensor baselines in the related goal of stress detection [30,31,32], suggesting feature-level or data-level fusion approaches as a starting point for this work.

A second challenge is that firefighter wellness ratings are inherently subjective and may be influenced by factors other than physiological or environmental states. For instance, firefighters and other workers doing physical labor may exhibit the “healthy worker effect” [33,34,35,36,37], where employment selection-bias makes them healthier on-average than the general population. Firefighters may under-report fatigue or distress due to occupational norms despite measurable physiological strain, an effect that has been observed in studies that use Borg’s Rating of Perceived Exertion (RPE) [38,39]. These norms may be reinforced by a workplace culture in which acknowledging fatigue or physical limitation can be perceived as a sign of weakness. Perceived wellness also reflects lifestyle factors such as sleep quality, cumulative fatigue, and mental burden [40,41,42].

A third related challenge is the high degree of personal variability in physiological data, which is exacerbated when limited data is available. Even if an individual is self-consistent in how they report perceived wellness, each person has different tolerances to stress which impact their perception in complex ways. Identical thermal and physical stressors will impact physiological responses differently across the population due to variation in fitness, age, heat acclimatization, and experience, just to name a few. Prior work has aimed to personalize wearable biomedical devices using machine learning or optimization methods on individual user data [43,44]. When per-user labeled data are not available, online and active learning approaches can personalize models to new individuals based on existing users with similar characteristics [45]. However, these methods perform worse than subject-specific classifiers and may fail in the presence of large deviations from existing data. There is a need for modeling approaches that can learn consistent trends across a small cohort and readily adapt to new incoming subjects.

This work addresses the challenges above with three main contributions. First, the multimodal wireless sensing system described above is validated on three challenging classification tasks: (1) distinguishing warm-up activities from live-fire suppression; (2) predicting whether a record is from a firefighter’s first or second training repetition; and (3) classifying a firefighter’s perceived wellness on a scale from 1–10. Second, a collection of evidence is provided showing that perceived wellness did not consistently track measured physiological stress in this experiment. Third, an LME approach is evaluated for one-label personalization, in which a new participant’s first wellness rating is used to calibrate predictions for their remaining phases. Overall, this study demonstrates the promise of wireless real-time data acquisition for firefighter monitoring and modeling approaches suitable for future large-scale validation.

## 2. Sensing System

The multimodal sensing system is designed to incorporate commercial wireless Bluetooth Low Energy (BLE) sensors in a portable, self-contained architecture intended for deployment at new sites. BLE was chosen for its widespread adoption in commercial sensors, low power consumption, and sufficient bandwidth for the relatively low data rates of the physiological and environmental signals. By using one modular data logger per firefighter and synchronizing to a central base station, it can aggregate data for use by fire captains with crews of varying sizes. As the system is fully battery-powered and requires no fixed infrastructure, it can be transported and set up at different training grounds or incident sites.

### 2.1. System Architecture

**Figure 1:**
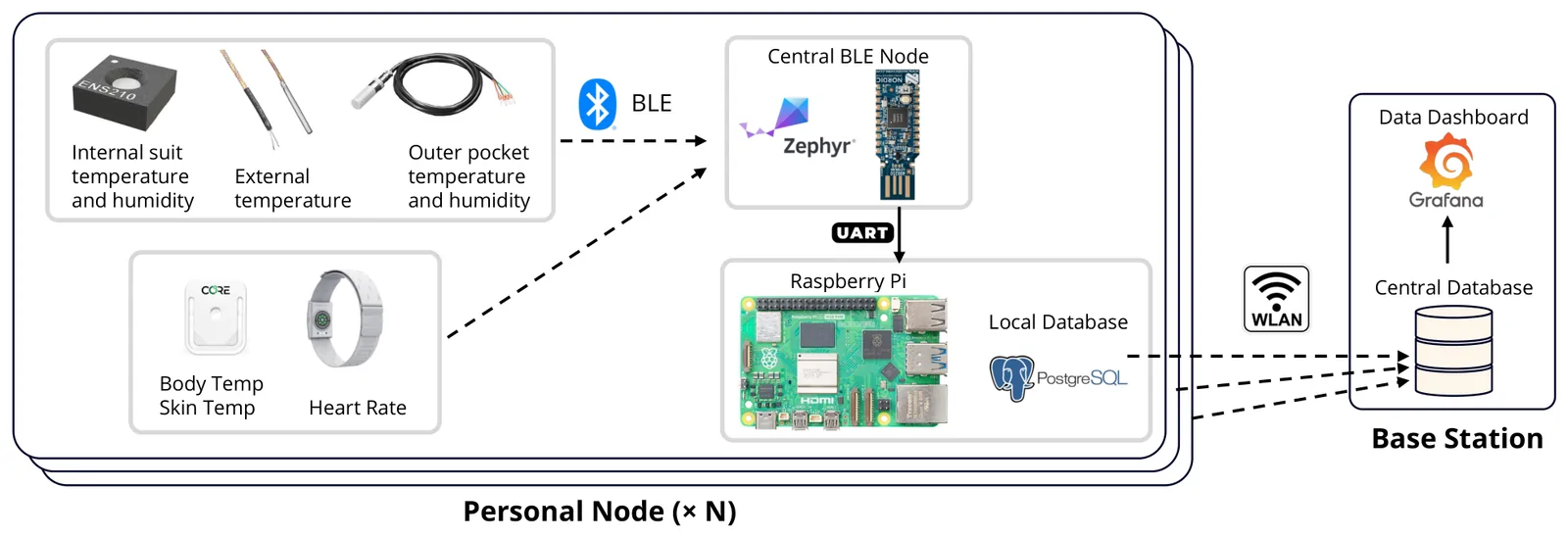
Overview of the sensing system architecture.

**Figure 2:**
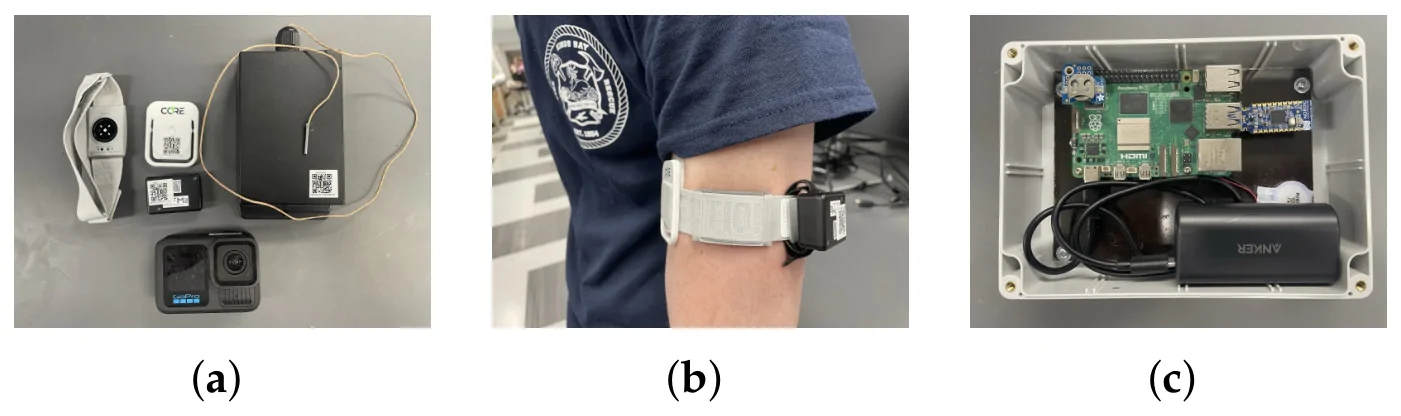
(**a**) Array of sensors. (**b**) CORE, SFM2, and COROS sensors fitted around the upper arm. (**c**) Logger node comprising a Raspberry Pi 5, Nordic Semiconductor BLE dongle, and Anker battery pack (Anker Innovations, Changsha, China) inside a NEMA-rated enclosure.

The sensing system (Figure 1) consists of several components: the sensors, the data logging node, and the central base station. Sensors worn by the participants (Figure 2a) continuously broadcast data over BLE, which is recorded by the data logging node also worn by the participant. A PostgreSQL database (version 16, PostgreSQL Global Development Group, www.postgresql.org) is hosted by each data logging node and stores the data locally to the device. A private WLAN network connects the data logging node to a central base station computer. Each firefighter carries a data logging node and broadcasts their own data to the central computer. Each independent database is aggregated into the central database on the central base station and made viewable during testing in real-time by a user interface and visualization tool (LabVIEW 2025 Q3, National Instruments, Austin, TX, USA, and Grafana 11.2.0, Grafana Labs, New York, NY, USA).

The purpose of the data logging node is to log and store data transmitted from each of the sensors worn by the firefighter. When a sensor sends a data notification, the data logger reads the data point, appends metadata (e.g., timestamp and sensor ID), and adds a row to the local database. The result is a single time history log for all sensor data worn by the firefighter during the activity. Sensor data is transmitted over BLE as unacknowledged notifications, so the logging node does not confirm receipt and individual data points may occasionally be dropped.

**Figure 3:**
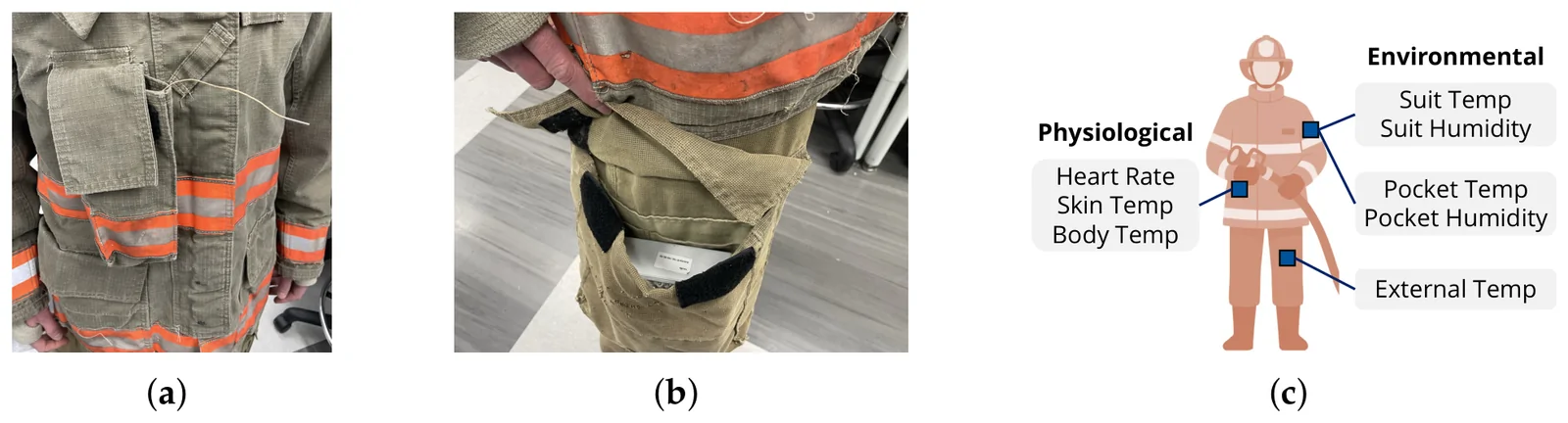
(**a**) Thermocouple probe in front pocket. (**b**) Data logging node worn in pant side pocket. (**c**) Firefighter sensors.

Each data logging node consists of a single-board computer running off a lithium-ion battery. The node has data storage and networking capabilities and is configured to run in a headless configuration. This implementation supports either a Raspberry Pi 5 or a Raspberry Pi Zero 2W (Raspberry Pi Foundation, Pencoed, Wales, UK). mounted inside an ABS-polycarbonate blend enclosure. The enclosure is NEMA and IP rated and also has a flame-resistant and self-extinguishing UL 94 5V rating. A BLE module connected through the node’s USB port is responsible for managing the BLE sensor connections. This module is the nRF52840 Bluetooth System-on-a-Chip (SoC) dongle from Nordic Semiconductor (Nordic Semiconductor ASA, Trondheim, Norway). and is programmed with custom software developed by the team in C using the nRF Connect SDK (version 2.6.0) and Zephyr OS (Zephyr Project, www.zephyrproject.org). The software manages the BLE connections with the sensors and aggregates the sensor information into a single data stream that is read, parsed, and logged by the data logging node. The node was worn by each participant in their pant side pocket, as shown in Figure 3b. Figure 3c shows the logger node in its packaging.

A logical replication schema is established between each data logging node and the central base station, which creates a publisher–subscriber model between the nodes and central computer. Because values are replicated rather than moved, a copy persists on each logging node, which adds redundancy for power or network loss to individual nodes and ensures no data is permanently lost. Synchronization to the base station occurs in real time whenever a node is within range of the private WLAN, and otherwise data accumulates in the local database and is replicated once connectivity is restored. This system uses a local, private network to easily transfer between test sites without relying on local internet services.

### 2.2. Sensors

The system is designed to accommodate a wide range of BLE commercial devices, including the wearable physiological and environmental sensors chosen for this study. All participants in this study wore the sensors listed in Table 1 and photographed in Figure 2, with their placement on the firefighter summarized in Figure 3c.

Commercial sensors are used to measure participant physiological characteristics. The COROS Heart Rate monitor is an optical heart-rate sensor that straps around the participant’s upper arm to measure heart rate, in beats per minute (Figure 2b). The Greenteg CORE sensor measures skin temperature and calculates internal core body temperature. This sensor is in contact with the participant’s skin on the upper arm and clips to the same strap as the heart rate monitor.

Firefighting turnout gear consists of several layers, including an outer shell, a moisture barrier, and a thermal barrier. To measure conditions inside the suit during activities, an environmental sensor measures the air temperature and relative humidity beneath all layers of the gear close to the firefighter’s body. The SFM2, a wearable IMU sensor by Sensor Maestros, which uses the ENS210 sensor for temperature and humidity sensing, is also attached to the upper arm strap (Figure 2b). All three arm-strap sensors are configured to transmit data at a rate of 1 Hz. To reduce the likelihood of sensors slipping off the participant’s arm during activities, a neoprene arm band is fit over the instrumentation and under the gear.

External environmental conditions are also measured. Participants are instrumented with K-type thermocouple probes in their turnout gear outer shell pocket (Figure 3a). The goal of this sensor is to measure the external temperature experienced by the firefighter. The sensor is placed in one of three locations depending on the available pocket space on the participant: a chest pocket, a waist pocket, or a leg side pocket. The sensing element, which has a fiberglass braid insulation and stainless-steel sheath, is left exposed to atmosphere, while the electronics are placed inside the pocket to protect from heat exposure.

These environmental channels are provided by a custom sensor node developed by the team, built around an Adafruit Feather Sense nRF52840 microcontroller board (Adafruit, Brooklyn, NY, USA). with a MAX31855 thermocouple signal conditioning amplifier (Analog Devices, Wilmington, MA, USA). The nRF52840 digitizes the thermocouple signal and transmits over BLE, integrating into the same architecture as the commercial BLE devices. The microcontroller also has an integrated board level temperature and humidity sensor (SHT-30) (Sensirion AG, Stäfa, Switzerland). which is collected in parallel with the thermocouple measurements. The board level measurement verifies the device remains at a safe operating temperature and also provides useful information about the external environmental conditions. These measurements are also configured to transmit data at 1 Hz.

## 3. Experimental Design

The Duke team conducted an experiment in January 2026 under an approved IRB protocol (Duke University 2023-0470) at Naval Submarine Base King’s Bay (NSBKB) with a total of 28 participating firefighters. The participants ranged widely in firefighting experience, from 0.5 years to 26 years (μ=7.78,σ=8.32, and median = 3.5 years). The experiment took place over two consecutive days, with 12 on the first day and 16 on the second day. Each firefighter was equipped with the suite of physiological and environmental sensors described in Section 2.2 and Table 1. These sensors recorded continuous time-series data as the firefighters completed an intensive training course meant to simulate a realistic fire scenario on a naval submarine.

Each trial consisted of two phases: a warm-up phase (WU) and a live-fire phase (LF). The training exercise uses two separate CONEX shipping containers. The first simulates the inside of a submarine using small portals and confined spaces. The second CONEX shipping container accommodates an enclosed live burning fire continually replenished by the operators of the training scenario. Participants completed the exact same training course twice—Trial 1 (T1) and Trial 2 (T2)—in pairs of two firefighters. Therefore, the experiment represents a repeated trials experiment with four sequential phases: Trial 1 Warm-Up (T1-WU), Trial 1 Live Fire (T1-LF), Trial 2 Warm-Up (T2-WU), and Trial 2 Live Fire (T2-LF).

**Figure 4:**
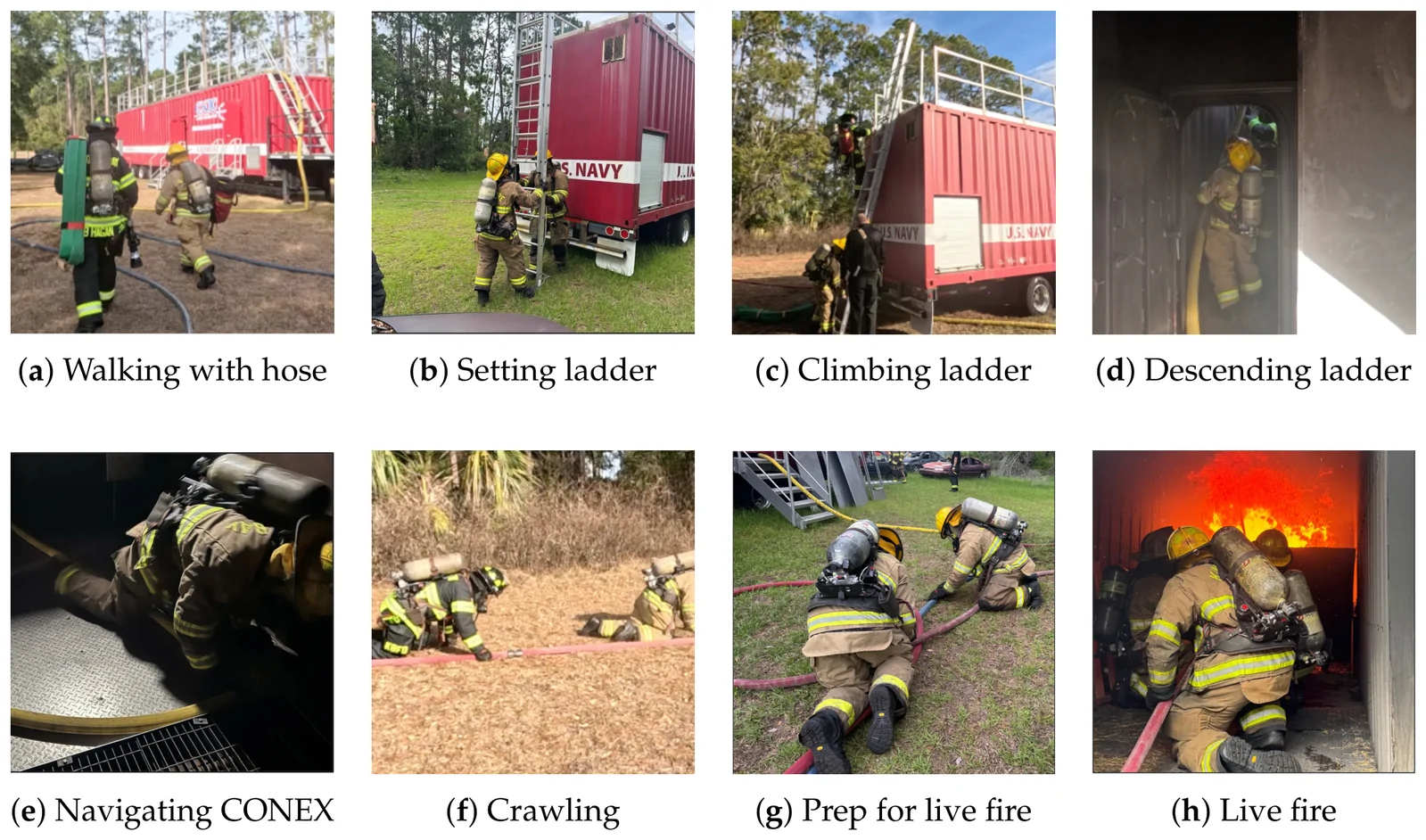
Chronological activities during one trial of the training exercise at Kings Bay. Environmental and physiological sensors recorded continuously throughout all phases.

The warm-up phases consisted of the following activities: carrying the fire hose and bag of rope to the first CONEX container (Figure 4a), setting a ladder (Figure 4b) and climbing up the ladder (Figure 4c), pulling up the hose with the rope, affixing the masks of the self-contained breathing apparatus (SCBA) while atop the container, crawling down the ladder into the container (Figure 4d), navigating through the container (Figure 4e), affixing the hose to a Y connector, exiting the container, and finally crawling along the hose line to the live-fire CONEX (Figure 4f). These exercises were meant to simulate what would happen during a submarine fire. For example, the crawling simulates being in a space filled with smoke where one needs to be as low to the ground as possible. Crawling along the hose line is also the primary means of navigating in and out of the ship, especially when there is no light.

Once they reached the second CONEX, they performed buddy checks and prepared for the live-fire phase (Figure 4g). This phase consisted of the participants entering the second CONEX with a burning fire (Figure 4h) and spending approximately five minutes inside with a lieutenant practicing different hose patterns on the fire. The live-fire phase concluded each trial, after which the participants would head to the recovery station. The only difference between the two trials was that the *leader* switched, i.e., the person in the front position of the pair of firefighters. The leader is considered the more intensive role in charge of important tasks during both warm-up (setting the ladder; affixing the hose to the Y connector) and live fire phases (guiding the hose while positioned closest to the fire).

Every participant answered a questionnaire before the start of the experiment which included the following fields: Age, height, weight, gender, PPE gear brand, Nomex hood brand, and time on the job as a firefighter, plus a series of subjective questions. These questions were as follows: “how well did you sleep last night (1–10, where 10 is the best)?”, “how much is on your mind? (1–10 where 10 is a lot on your mind)”, “what is your energy level? (1–10 where 10 is most energetic)”, “do you consume caffeine? (Y/N), If so, how much in the last 24 h?”, and “Do you consume nicotine? (Y/N), If so, how much in the last 24 h?”.

All participants also had their vitals measured by a fire department medic, including the following: blood pressure, heart rate, blood oxygen, respiratory rate, CO level, and cylinder PSI of the oxygen tank of their SCBA. These measurements occurred before starting the training circuit as well as in the recovery phase after each trial (3 times total).

The participants answered a series of self-assessment questions immediately after each of the four phases. These questions were assessed on a scale from 1 to 10, with 10 being the best: “How well do you feel?”, “How were your communications?”, “How was your speed?”, “How was your respiratory control?”, “How was your temperature comfort?”, and “How was your overall performance?”. The primary target variable in this study was chosen to be the first question. It served as a momentary summary of the firefighter’s state after each phase, whereas the remaining questions assessed more specific aspects of the exercise. This is consistent with U.S. Fire Administration rehabilitation guidance, which recommends considering vital signs together with signs of injury or distress and talking with firefighters during evaluation, rather than relying on vital signs alone [46]. Accordingly, the target is referred to as self-reported wellness, which is distinct from a measure of fatigue or physiological health. The results of the self-assessment and analysis relating to the perceived wellness rating are presented in Section 6.2.

## 4. Methods

### 4.1. Preprocessing

Raw sensor data undergo preprocessing prior to modeling and analysis. First, outliers are removed in the external temperature (e.g., erroneous readings greater than 500 °C) and body/skin temperature (e.g., readings equal to 0 °C, which represent signal loss) modalities. Next, the continuous time histories are segmented by phase (e.g., T1-WU, T1-LF, T2-WU, and T2-LF) using timestamps collected manually during the experiment. Each phase-segmented multimodal time history represents a single “observation.”

Although all 28 participants completed the four experimental phases, sensor failures and insufficient sensor coverage meant that some phase recordings could not be used. Two participants were excluded because both body- and skin-temperature streams were unavailable, leaving 26 participants and 104 possible phase observations. Six observations were unavailable because Trial 2 sensor data were missing for three participants, and eleven additional phase recordings contained no usable data or did not meet a 30% overall-coverage requirement. The final dataset therefore contains 87 phase-level observations from 26 participants, with an average of 3.3 phases per participant:

- A total of 18 of 26 participants have all 4 phases (complete data);
- A total of 2 participants have 3 phases;
- A total of 3 participants have 2 phases;
- A total of 3 participants have only 1 phase.

Because the 30% threshold was applied to overall multimodal coverage rather than to each sensor channel individually, a retained phase could still lack usable data from an individual sensor. Of the phase-level sensor summaries expected across the 87 retained observations, 4.2% were unavailable. Eleven observations (12.6%) were missing at least one of the ten predictors used in the reported wellness models. Within each LOSO fold, missing phase-level predictors were imputed using the corresponding feature means calculated only from the outer training set before standardization. These training-set means were also used to impute missing predictors for the held-out participant, as detailed in Algorithm 1. Because BLE notifications were unacknowledged and no packet-level acknowledgment log was collected, these values describe observed sensor missingness rather than a confirmed BLE-specific packet-loss rate.

**Figure 5:**
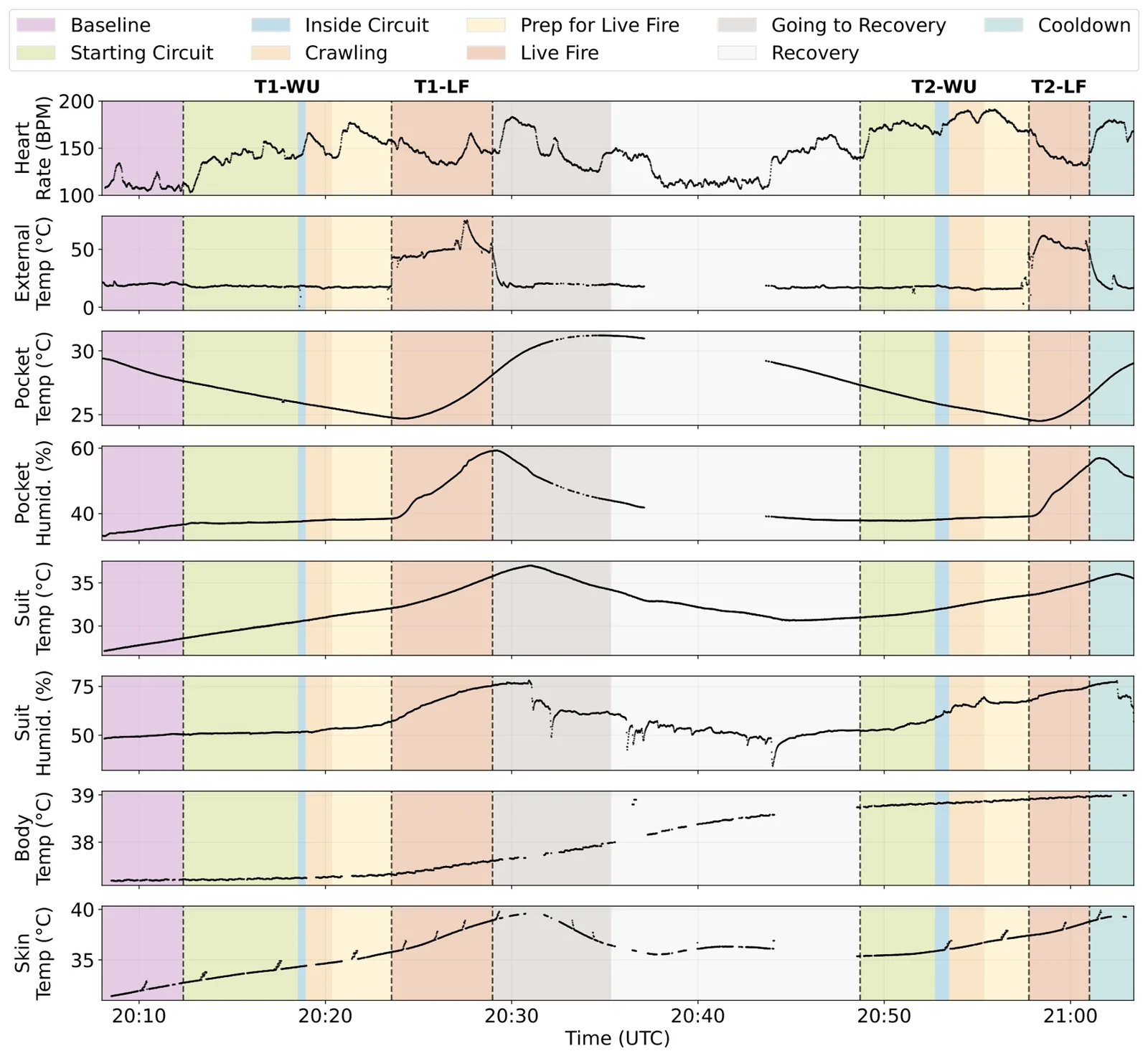
Complete multi-sensor time history for a representative participant. Color-coded vertical bands indicate different activities in the training loop. The four phases are segmented using dotted lines and labels at the top of the plot.

Baseline physiological and environmental values are computed for each participant from a period of downtime prior to the experiment (Figure 5). Because the duration of this downtime varies on a person-by-person basis depending on the status of the experiment and readiness of the research team, the baseline period is cropped to the 5 min directly before beginning the first training circuit. The baseline mean and standard deviation are then computed for each sensor type.
**Algorithm 1** Elastic Net LOSO pipeline.**Require:** Dataset $D={\left\{\left(x_{i},y_{i},s_{i}\right)\right\}}_{i=1}^{n}$, participant set S, candidate features F  1:**for all** s∈S **do**  2:      $D_{\mathrm{train}}\leftarrow \left\{\left(x_{i},y_{i},s_{i}\right)\in D:s_{i}\neq s\right\}$  3:      $D_{\mathrm{test}}\leftarrow \left\{\left(x_{i},y_{i},s_{i}\right)\in D:s_{i}=s\right\}$  4:      $\mu_{s}\leftarrow$ column means of $D_{\mathrm{train}}\left[F\right]$  5:      $\left(X_{\mathrm{train}},X_{\mathrm{test}}\right)\leftarrow \left(D_{\mathrm{train}}\left[F\right],D_{\mathrm{test}}\left[F\right]\right)$ with missing entries replaced by $\mu_{s}$  6:      $\sigma_{s}\leftarrow$ column standard deviations of $X_{\mathrm{train}}$  7:      $X_{\mathrm{train},f}\leftarrow \left(X_{\mathrm{train},f}-\mu_{sf}\right)/\sigma_{sf}$ for every f∈F  8:      $X_{\mathrm{test},f}\leftarrow \left(X_{\mathrm{test},f}-\mu_{sf}\right)/\sigma_{sf}$ for every f∈F  9:      $y_{\mathrm{train}}\leftarrow$ outcome vector from $D_{\mathrm{train}}$10:      $M_{s}\leftarrow \mathrm{ElasticNetCV}\left(X_{\mathrm{train}},y_{\mathrm{train}},\mathrm{cv}=5\right)$11:      ${\hat{y}}_{s}\leftarrow M_{s}\left(X_{\mathrm{test}}\right)$12:      ${\hat{\beta }}_{s}\leftarrow$ coefficient vector of $M_{s}$13:      $z_{sf}\leftarrow 1$ if ${\hat{\beta }}_{sf}\neq 0$, and $z_{sf}\leftarrow 0$ otherwise, for every f∈F14:**end for**15:**return** held-out predictions, coefficients, and non-zero-coefficient indicators

### 4.2. Featurization of Time-Series Data

Summary statistics are computed over each complete phase for each sensor channel for downstream modeling usage. The seven aggregate features are as follows: *mean, standard deviation, minimum, maximum, range* (max–min), as well as the following:

- *slope*: The ordinary least-squares slope of sensor readings regressed against their sample index, i.e., ${\hat{\beta }}_{1}$ from $y_{i}=\beta_{0}+\beta_{1}i+\epsilon_{i}$, where i=0,1,…,n−1. Observations with fewer than 10 data points (e.g., dropped data) are assigned NaN slope.
- *delta*: The difference between the sensor mean in the current observation and the subject’s baseline mean for the same sensor channel. Captures how much sensor measurements deviate from the subject’s resting state.

Consequently, prediction is evaluated retrospectively at the phase level. A sliding window approach may be considered for future studies with a larger magnitude of data for training real-time prediction models with the same features.

### 4.3. Statistical Analysis

The experiment in this study uses a repeated-measures design where each participant completes two repetitions of both the warm-up phase and the live-fire phase, for a total of (up to) four observations (phases) per subject. The perceived wellness score (1–10) has an empirical distribution which is not normally distributed (Section 6.2 below). It is assumed to be an ordinal variable. Hypothesis tests are used to evaluate the significance of differences in data features, wellness scores, and model results across experimental phases. Non-parametric tests including the Wilcoxon signed-rank test are preferred here for paired data which is not guaranteed to be normally distributed across the population. Spearman’s rank correlation is preferred over Pearson correlation for relationships that are not guaranteed to be linear. Statistical significance is evaluated at α=0.05, but caution is exercised in drawing conclusions from these tests due to the small sample size.

## 5. Models

As discussed in the introduction, this study considers multiple different prediction tasks related to firefighter wellness and activity understanding. Here, the models for each downstream prediction task are introduced. Logistic Regression (LR) classifiers are used to distinguish a firefighter’s warm-up activities from live-fire suppression (Section 6.1.2) as well as predict whether a sensor record is from a participant’s first or second training repetition (Section 6.1.3). An Elastic-Net (EN) model is chosen to predict firefighter wellness on a scale from 1–10 (Section 6.3) thanks to its inherent feature-selection and ability to handle correlated features. Finally, a linear mixed-effects model extends the wellness score regression to personalize predictions to individual firefighters (Section 6.3.2). All models take as input the standardized summary-statistic features defined in Section 4.2 and are evaluated using Leave-One-Subject-Out (LOSO) cross-validation. Details of the models are provided below.

### 5.1. Logistic Regression Classifiers

Logistic Regression (LR) models are used to predict the activity class from the sensor and environmental features for the classification problems described in Section 6.1.2 and Section 6.1.3. LR classifiers model the probability of class membership from 0 to 1 using the sigmoid function applied to a linear model. For binary (2-class) classification, the model estimates $P\left(y=1|x\right)=\sigma \left(x^{⊤}\beta +\beta_{0}\right)$, where $\sigma \left(z\right)=1/\left(1+e^{-z}\right)$ is the sigmoid function, and $\beta_{0}$ is a scalar intercept term. LR is adapted for multi-class problems using the “one-vs-rest” strategy, which trains *K* independent binary classifiers that each distinguish one class from the remaining K−1 classes. All LR models in this study use $\ell_{2}$ regularization with C=1.0. Performance is evaluated using participant-level Leave-One-Subject-Out (LOSO) cross-validation. Within each fold, missing-value imputation and feature standardization are fit using only the remaining participants before predictions are generated for all phases from the held-out participant.

### 5.2. Wellness Score Prediction Using Elastic-Net

The Elastic Net [47] (EN) is used to predict the perceived-wellness score from the standardized sensor, environmental, and demographic features. It is a penalized linear regression method that uses the ordinary least-squares loss with a weighted mixture of both $\ell_{1}$ (Lasso) and $\ell_{2}$ (Ridge) regularization. The $\ell_{1}$ term drives certain coefficients exactly to zero, performing implicit feature selection of more useful features. The $\ell_{2}$ term prevents total elimination of variables that may be closely correlated, which may be undesirable when using highly-correlated features such as participant background demographic information. The parameters β are estimated as follows:

(1) $$\hat{\beta }=\underset{\beta }{\mathrm{argmin}}\left({∥y-X\beta ∥}^{2}+\lambda_{2}{∥\beta ∥}^{2}+\lambda_{1}{∥\beta ∥}_{1}\right),$$

where y is the vector of perceived-wellness scores, X is the corresponding matrix of standardized features (Section 4.2), and $\lambda_{1}\geq 0$ and $\lambda_{2}\geq 0$ govern the strength of the $\ell_{1}$ and $\ell_{2}$ penalties, respectively. Intuitively, a larger $\lambda_{1}$ yields a sparser model with fewer active features, while $\lambda_{2}$ stabilizes the coefficients of correlated predictors. EN models are fit using scikit-learn’s ElasticNetCV [48]. To evaluate one participant, all observations from that participant are first set aside. ElasticNetCV selects the penalty parameters by *k*-fold cross-validation (k=5) using the remaining participants’ data. The model with the selected hyperparameters is refit using all observations from the remaining participants and then evaluated on the set-aside participant. Thus, the participant being evaluated does not contribute to imputation, standardization, hyperparameter selection, coefficient estimation, or fold-specific feature selection for that LOSO fit. The complete procedure is summarized in Algorithm 1.

After completing all LOSO folds, features are ranked by their cross-fold survival rate, defined as the proportion of folds (subjects) in which a particular feature had a non-zero coefficient (i.e., the indicator $z_{sf}=1$). The mean absolute coefficient magnitude is used to break ties. LOSO performance is then compared for the top *N* ranked features, where *N* ranges from 5 to 65 in increments of 5. Because this ranking aggregates results from all LOSO folds, the feature selection is not fully independent of each held-out participant. Therefore, this top-*N* comparison should be viewed as an exploratory descriptive analysis rather than an unbiased estimate of performance for a locked predictive pipeline. In the event that additional future data is collected, the selected features may serve as a starting point for a fully independent future validation.

### 5.3. Mixed Effects Model

Consider the standard linear mixed-effects (LME) model for repeated measures [49,50]:

(2) $$y_{ij}=x_{ij}^{⊤}\beta +u_{i}+\varepsilon_{ij},$$

where $y_{ij}$ is the wellness rating for subject *i* in phase j∈{1,2,3,4}, $x_{ij}$ is the feature vector, β is the vector of fixed-effect coefficients shared across all subjects, $u_{i}∼N\left(0,\sigma_{u}^{2}\right)$ is a subject-specific random intercept capturing the individual’s deviation from the population mean, and $\varepsilon_{ij}∼N\left(0,\sigma_{e}^{2}\right)$ is within-subject residual noise. The random intercept $u_{i}$ and residual $\varepsilon_{ij}$ are assumed independent.

The fixed effects β capture population-level relationships that are expected to relate the features to wellness across subjects, e.g., higher body temperature being associated with lower wellness (note: conceptual example, not a real result). The random intercept $u_{i}$ captures a firefighter-specific component that is constant across all phases for that participant. It is intended to capture offsets whereby some individuals consistently rate their wellness higher or lower than the population model would predict, due to individual differences in pain tolerance, baseline fitness, or psychological factors. The variance terms $\sigma_{u}^{2}$ and $\sigma_{e}^{2}$ are estimated by Restricted Maximum Likelihood (REML) [51].

After training the model on all but the held-out subject, now suppose that the held-out new subject *i* completes the first phase of the experiment (T1-WU). This provides the observation $y_{i1}$. The fixed-effects prediction for this phase is ${\hat{y}}_{i1}=x_{i1}^{⊤}\hat{\beta }$, and the residual is $r_{i1}=y_{i1}-{\hat{y}}_{i1}$. This residual reflects both the subject’s random intercept $u_{i}$ and the observation noise $\varepsilon_{i1}$. The Best Linear Unbiased Prediction (BLUP) [52] of the random intercept given $n_{i}$ observations from subject *i* is as follows:

(3) $${\hat{u}}_{i}=\frac{\sigma_{u}^{2}}{\sigma_{u}^{2}+\sigma_{e}^{2}/n_{i}}\cdot {\bar{r}}_{i},$$

where ${\bar{r}}_{i}=\frac{1}{n_{i}}\sum_{j=1}^{n_{i}}r_{ij}$ is the mean fixed-effect residual for subject *i*. The term $\lambda \left(n_{i}\right)=\sigma_{u}^{2}/\left(\sigma_{u}^{2}+\sigma_{e}^{2}/n_{i}\right)$ is the *shrinkage factor*, which controls how much of the observed residual is attributed to the subject’s true intercept versus noise. With a single calibration observation ($n_{i}=1$), the shrinkage factor simplifies to [53]:

(4) $$\lambda \left(1\right)=\frac{\sigma_{u}^{2}}{\sigma_{u}^{2}+\sigma_{e}^{2}}=\mathrm{ICC},$$

yielding ${\hat{u}}_{i}=\mathrm{ICC}\cdot r_{i1}$, where ICC is the Intraclass Correlation Coefficient by definition for the model given in Equation (2). Note that if multiple repeated observations become available for subject *i*, then the estimation of the random intercept becomes closer to $r_{i}$ as $n_{i}$ increases.

Once the BLUP ${\hat{u}}_{i}$ is estimated, subsequent predictions for subject *i* can be calibrated by the results of their first perceived wellness rating (the target variable, scale from 1–10):

(5) $${\hat{y}}_{ij}^{\mathrm{cal}}=x_{ij}^{⊤}\hat{\beta }+{\hat{u}}_{i},\;j=2,3,\ldots$$

This shifts the prediction from the population mean toward the subject’s inferred personal baseline. The magnitude of the shift is governed by the data-derived shrinkage factor.

Fitting an LME model (Equation (2)) with too many fixed-effects features is prone to convergence difficulties with REML optimization [51]. Therefore, the set of features identified by the retrospective EN analysis (Section 5.2) are used as fixed effects. Within each LOSO fold, missing values are filled and features are standardized using the training subjects only. Then, a feature-adjusted LME model is fit on the *training subjects only* to estimate $\hat{\beta }$, ${\hat{\sigma }}_{u}^{2}$, and ${\hat{\sigma }}_{e}^{2}$ via REML. This yields the fold-specific ICC shrinkage factor. After observing the new subject’s first wellness score, the residual $r_{i1}=y_{i1}-{\hat{y}}_{i1}$ is calculated, and the BLUP ${\hat{u}}_{i}=\hat{\lambda }r_{i1}$ is applied to subsequent predictions.

In summary, the LME model acknowledges that personal differences in wellness perception should be modeled separately from population-level fixed effects. Using a one-label personalization setting, the held-out firefighter contributes no data to model fitting, imputation, or standardization, but their true T1-WU wellness rating is subsequently observed and used to calibrate predictions for their remaining phases.

### 5.4. Evaluation

Ordinarily, models would be evaluated using *k*-fold cross-validation. However, randomly dividing observations into *k* folds would allow the model to observe some examples from a particular test subject during training, which is considered a form of data leakage. Instead, model fits in this paper use LOSO cross-validation, where training data comprises all observations from n−1 subjects, and test data contains all observations from the held-out subject. The evaluation is repeated for each of the n=26 subjects in this study, with results averaged over all folds. The subsequent cross-fold feature-survival ranking and reduced-feature comparison are described separately in the EN pipeline above.

The classification tasks in Section 6.1.2 and Section 6.1.3 use standard metrics including accuracy, precision, recall, F1 score, and the area under the precision-recall curve (AUPRC). Confusion matrices are also presented to visualize per-class error patterns. Self-reported wellness is an ordinal rating on a discrete 1–10 scale, so both regression and classification metrics are appropriate. In addition to confusion matrices, the following metrics are reported:  

- Accuracy: the fraction of predictions whose rounded value matches the true wellness exactly.
- Adjacent accuracy: the fraction of predictions within ±1 of the true wellness. This is relevant as adjacent ratings (e.g., 7 vs. 8) are often interchangeable in practice.
- Mean absolute error (MAE): the average absolute difference between the continuous prediction and the true integer wellness rating.

All metrics are computed per subject (i.e., per LOSO fold) and reported as mean ± standard deviation across the folds. For LOSO prediction-performance metrics and paired between-model MAE differences, 95% confidence intervals are estimated using a participant-level non-parametric percentile bootstrap with 10,000 resamples. Repeated phase observations and paired model errors are kept together within each resampled participant.

## 6. Results

### 6.1. Validation of Multimodal Sensor System

This section evaluates the complementary roles of physiological and environmental measurements in characterizing activity context and firefighter response. As discussed in Section 1, among the few studies that incorporate both wearable and environmental sensors, none explicitly examine how environmental measurements can contextualize physiological responses in firefighters.

#### 6.1.1. Example Data

Figure 5 shows the full sensor time history for a representative participant across both trials. These time series contain useful information for the task at hand. External temperature spikes suddenly upon the firefighter entering the live-fire CONEX and remains elevated (reaching as high as 60 °C) until exiting, at which point it quickly returns to baseline. The humidity inside the protective gear reaches nearly 80% during the live-fire phase, likely due to the extinguishing exercises using large volumes of water in the closed environment, as well as perspiration and the evaporation of sweat. In contrast, heart rate increases to 150–170 bpm and remains elevated during most of the duration of each trial. For this firefighter, the heart rate is *higher* on average during WU phases compared to LF phases. Body temperature rises monotonically from ∼37.3 °C to ∼38.5 °C over the course of both trials, which may reflect cumulative thermal strain or the specific response profile of the CORE sensor. Together, these measurements provide different types of context. Environmental signals mark exposure to the live-fire environment, while physiological signals describe the firefighter’s response over the repeated trials.

#### 6.1.2. Phase Classification: Warm-Up (WU) or Live Fire (LF)

**Figure 6:**
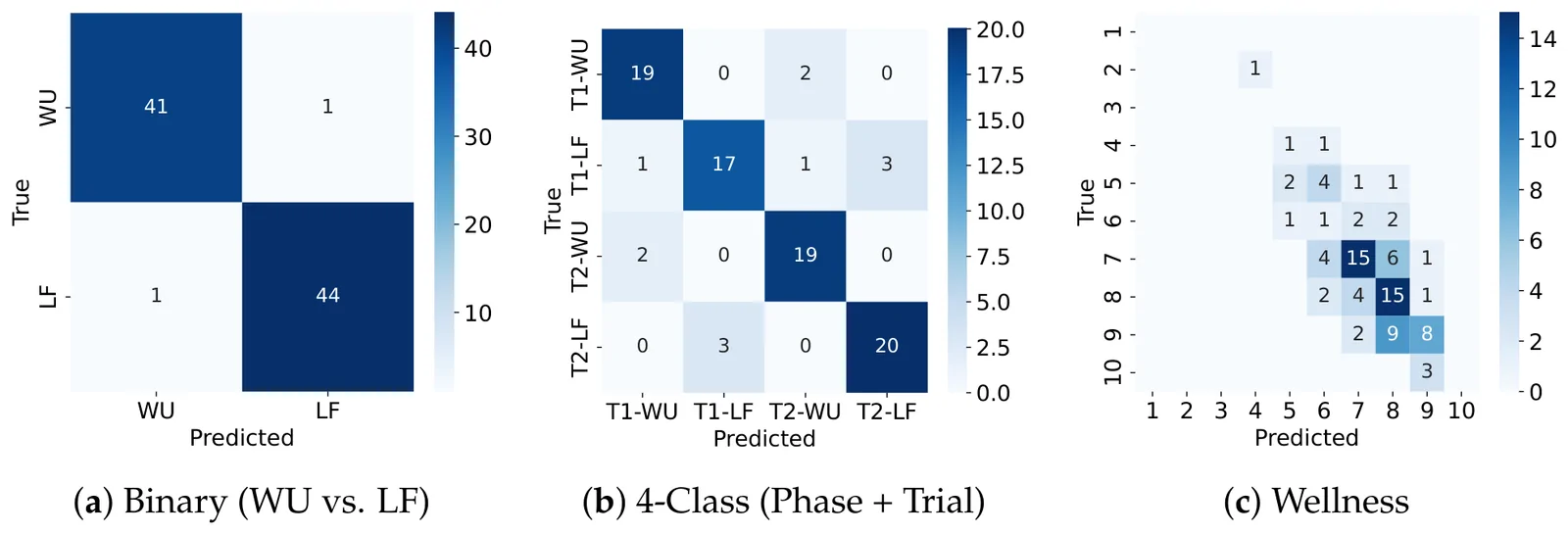
Confusion matrices for different classification tasks. (**a**) Binary warm-up vs. live-fire classification. (**b**) Four-class phase classification. (**c**) Elastic Net wellness prediction on the 1–10 rating scale.

First, a simple logistic regression model is trained to assess how well the sensor features classify whether a firefighter is participating in WU vs. LF phases. Three separate feature sets are tested based on different combinations of sensors (refer to Table 1): the physiological sensors only, the environmental sensors only, and all sensors. In each case, every sensor modality is featurized across the duration of the phase using the seven statistical features defined in Section 4.2, for a total of up to 56 features. The results are illustrated in Figure 6a and Table 2. Under participant-level LOSO cross-validation, the classifiers using all sensors and environmental sensors only both correctly classify 85 of 87 held-out phase observations (97.7%). For the all-sensor model, one of 45 live-fire observations is classified as warm-up and one of 42 warm-up observations is classified as live fire, corresponding to recalls of 97.8% and 97.6%, respectively. The classifier using only physiological sensors is able to classify the phase type to a lesser extent, with 82.8% accuracy. These results suggest that environmental sensors were more suitable than physiological sensors for detecting proximity to heat and fire in this study, as would be expected. Accordingly, this controlled binary task primarily validates environmental context sensing; it does not demonstrate that adding physiological features improves phase-classification accuracy.

To understand why, Table 3 presents the results of Wilcoxon signed-rank tests performed on the mean-value features of each sensor modality, comparing paired WU and LF observations for each subject. All five environmental modalities show differences between the WU and LF phases, helping explain why a model with only these features can perform well. The temperature- and humidity-related measurements all trend higher in LF phases. Skin temperature, which is a physiological measurement, shows the same trend. Heart rate and body temperature are also different between the two phase types, but with smaller effect sizes. Notably, heart rate is higher on average during warm-up phases than during LF phases. Wellness scores are also higher in the LF phase (p=0.0001). Because environmental measurements reliably identify the heat stress context, the next section investigates whether physiological sensors provide additional utility in tracking exertion across repeated trials.

#### 6.1.3. Phase and Trial Classification

Next, a second classification model is trained to simultaneously infer both the phase type and the trial number as a 4-class prediction (T1-WU, T1-LF, T2-WU, and T2-LF). The trial number was conceived as as an exploratory proxy for cumulative fatigue over time, since both trials occurred sequentially using the same sensors in roughly the same environmental conditions. However, the results should be interpreted as trial-repetition classification rather than fatigue detection, as potential confounders include accumulated heat, task familiarity, recovery effects, sensor drift, and differences in the tasks executed. The model is a logistic regression classifier (Section 5.1). Figure 6b and Table 4 show the results. In contrast to the simpler two-class problem, the classifier that uses all sensor modalities performs the best, showing an accuracy improvement of 8% over the model that uses only environmental sensor data, which itself has a 10.3% accuracy gain over the physiological-only model. The confusion matrix in Figure 6b shows that the multimodal model misclassifies 12 observations out of 87. Out of the 12 errors, only 2 are misclassified by phase type.

**Figure 7:**
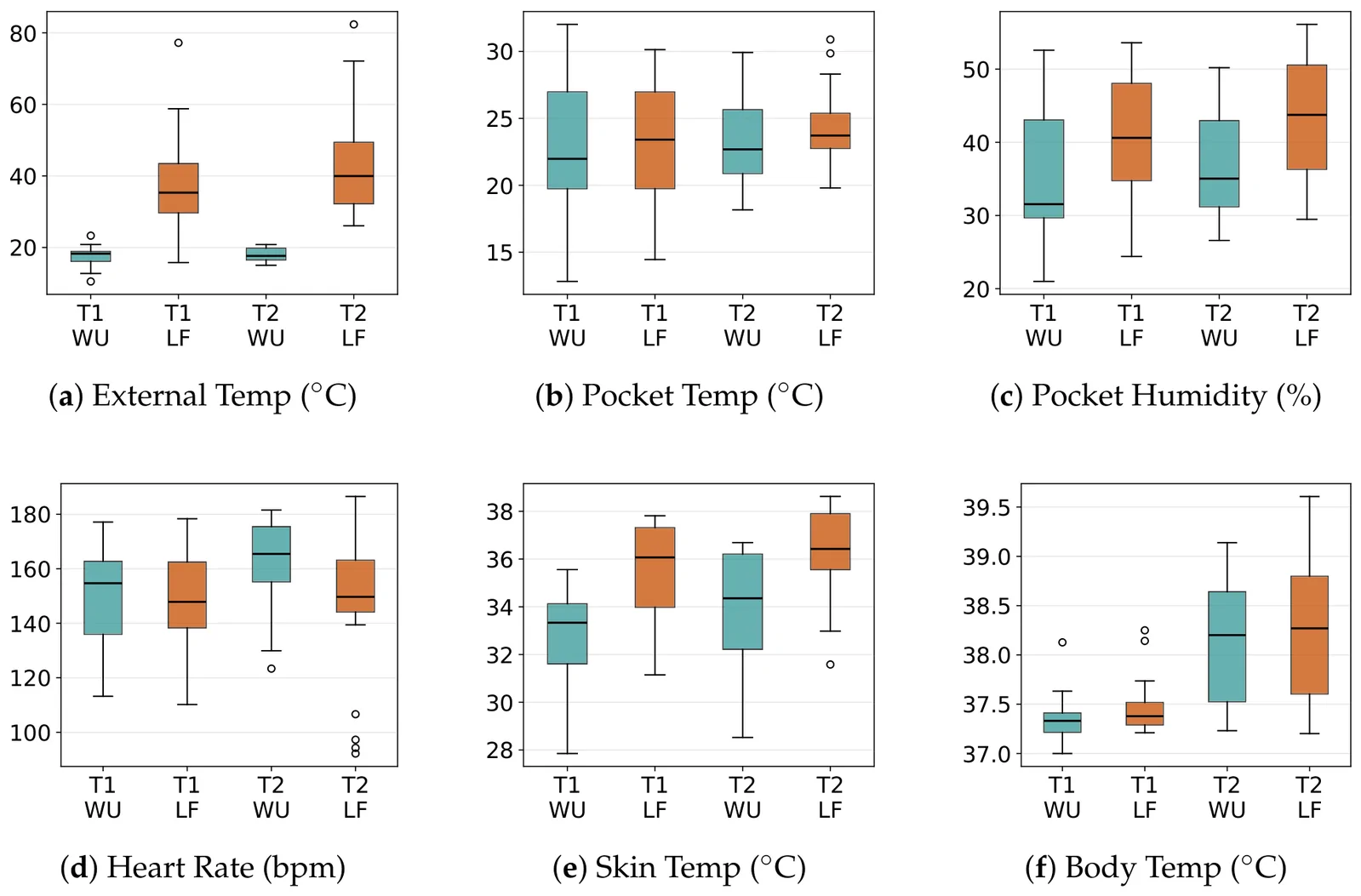
Sensor readings by phase type and trial. Teal boxes indicate warm-up phases; orange boxes indicate live-fire phases. Circles denote outlier data points.

Figure 7 visually displays the means of different sensor modalities by phase. Figure 7a suggests that external temperature alone may be sufficient to discriminate between WU and LF phases during controlled test environments similar to the current experiment. Figure 7f reveals an increasing average trend in body temperature from the start of recording to the end, with each successive phase higher than the preceding. Additionally, Table 5 presents the results of Wilcoxon signed-rank tests to understand whether the sensor-modality means are significantly different for each specific subject from T1 to T2. The tests reveal differences between the T1-WU and T2-WU in the mean and standard deviation features of heart rate as well as the mean body temperature. Given the variation in physiological features between trials, these results help explain why the 4-class classification problem is assisted by physiological sensors more than in the 2-class problem.

Overall, the evidence supports two primary conclusions for the present experiment: (1) environmental measurements of temperature and humidity are more informative than physiological measurements for identifying whether a firefighter is in close proximity to a fire or conducting more intense physical activity; and (2) physiological measurements are informative to understand how many times firefighters have performed the training activity, which may be useful information for other downstream predictive tasks.

### 6.2. Subjectivity of Perceived Wellness

The primary target variable in this study is the firefighter response to the question: “On a scale from 1–10, how well do you feel?” As described in Section 3, this question was asked to each firefighter before starting the training as well as immediately after each of the four phases. This section explores the wellness scores in detail and shows that they did not consistently track measured physiological indicators in this experiment.

#### Target Variable Statistics

**Figure 8:**
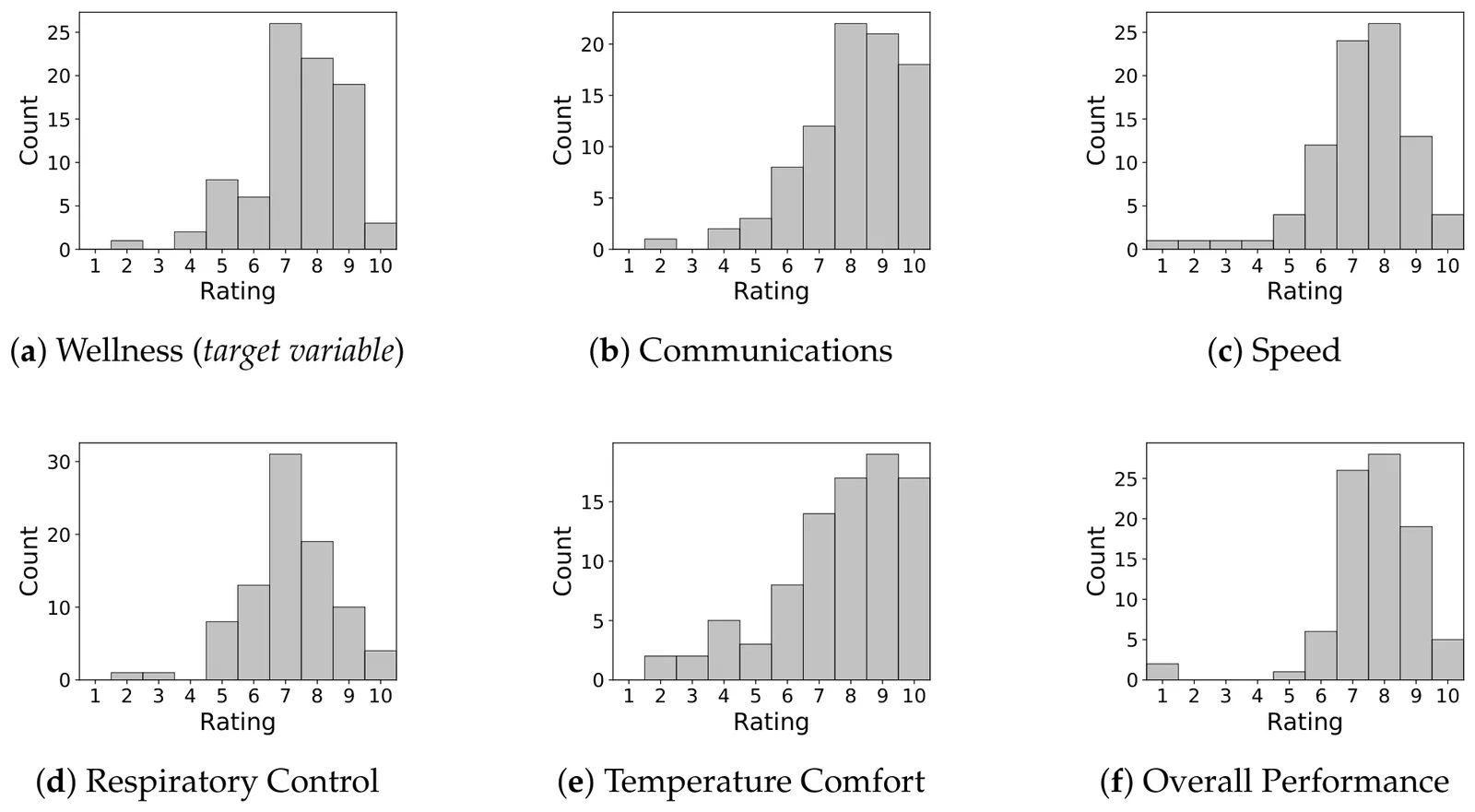
Distribution of self-reported wellness and related survey dimensions (1–10 scale). Wellness ratings are concentrated in the 7–9 range, with mean = 7.41 and std = 1.48.

**Figure 9:**
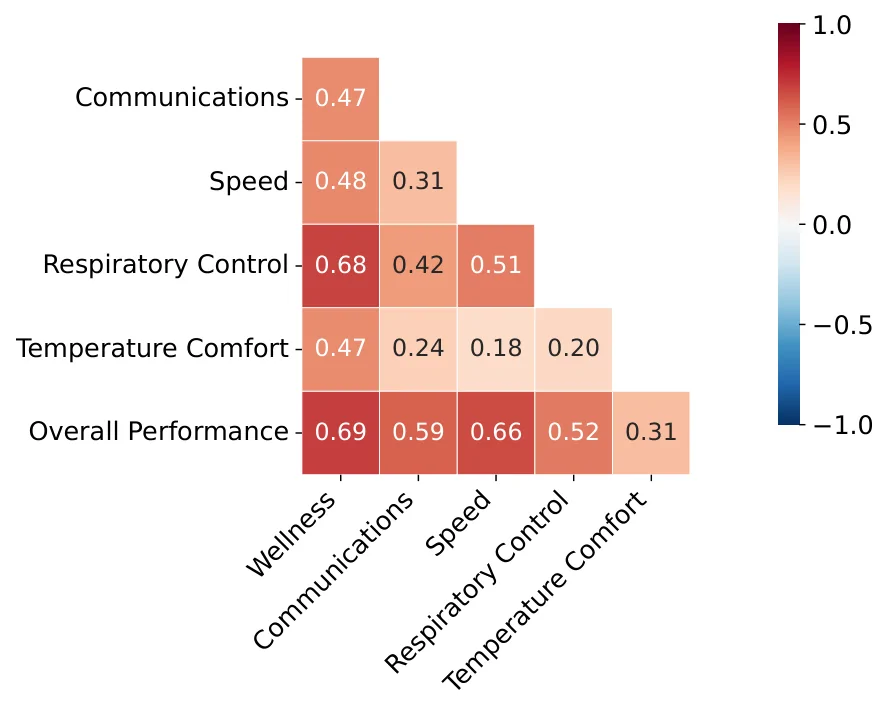
Spearman rank correlations between dimensions of the self-assessment survey. Wellness correlates most strongly with overall performance (0.69) and respiratory control (0.68).

Figure 8 shows the aggregated results of the self-assessment survey presented to firefighters after each phase of the training exercise (refer to Section 3). The results reveal that all responses are centered in the upper half of the scale. For the primary target variable, wellness, no participants reported the minimum wellness score of 1 in any of the phases. The target variable therefore has an imbalanced distribution. Figure 9 reveals that wellness correlates most strongly with respiratory control and overall performance and least strongly with temperature comfort and communications. These correlations are viewed as descriptive relationships that held in this experiment and may worth validating in future controlled studies.

**Figure 10:**
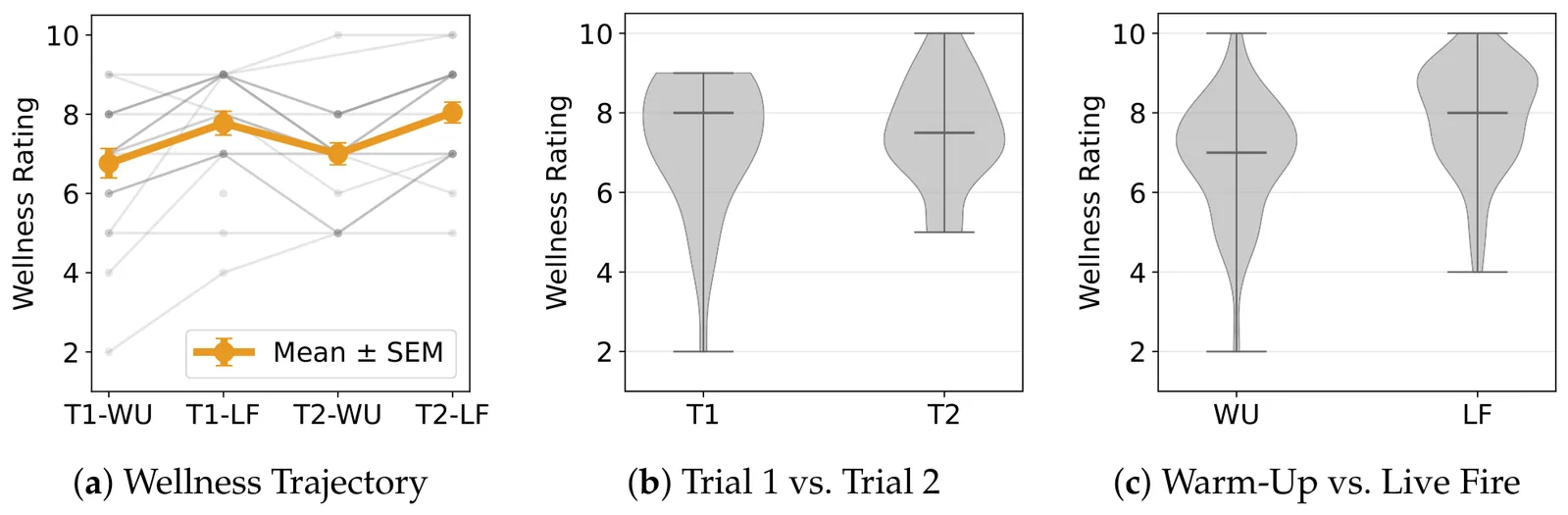
Wellness ratings across experimental phases. (**a**) Individual trajectories (gray) with population mean ± SEM (orange, thicker). (**b**) Trial 1 (μ=7.35±1.43) vs. Trial 2 (μ=7.53±1.28). (**c**) Warm-Up (μ=6.91±1.34) vs. Live Fire (μ=7.96±1.15).

Since the wellness scores occupy the upper half of the scale overall, it is also useful to see how they vary over time. Figure 10a plots each firefighter’s trajectory of wellness scores in gray, with the mean trend line across participants overlaid as a bold orange line. The other two subfigures, Figure 10b,c, show violin plots of the distributions of responses divided by trial and by phase type, respectively. The score distribution for WU phases is centered lower than for LF phases (p=0.0001, Table 3). The score distribution for T1 is lower on average than for T2 in this experiment, although this is not statistically significant per the Wilcoxon signed-rank test (W=44, p=0.5629). The figures show that the highest average wellness rating occurred immediately after the final phase (T2-LF). The perceived wellness increased, on average, after participating in two trials instead of just one. Importantly, Table 5 shows that while wellness increased from T1 to T2 during WU phases, so did heart rate and body temperature. This finding is contrary to expectations, as one would expect that cumulative fatigue over the duration of the experiment leads to lower wellness. The results support the observation that self-reported wellness did not consistently track the measured physiological indicators in this experiment.

A possible explanation is that occupational norms, adrenaline, relief after completing a rigorous task, or other unknown factors may skew self-reporting. It is worth noting that participants were informed in advance that they would participate in two trials. Therefore, prior knowledge of the experimental procedure could introduce bias by allowing firefighters to mentally prepare for two sequential periods of exertion. Additional randomized experiments would be necessary where different groups perform different numbers of repeated trials, with and without prior knowledge, to further explore this result in a controlled setting.

To assess whether participant demographic variables are associated with the perceived wellness, a linear mixed-effects model is fit with random intercepts for each participant (Table 6). Each continuous predictor is transformed to z-score coordinates first in order to standardize the feature scales. The model shows that age, experience, and height are significantly associated with wellness score. Experience and height are positively associated with wellness, while age is negatively associated. Age and experience themselves are positively correlated (Spearman’s rank correlation ρ=0.795), but they act in opposite directions. However, the results should be interpreted with caution given the wide confidence intervals and small number of observations in this study.

Since demographic factors seem to contribute to perceived wellness, a null mixed-effects model is used to compute the ICC (Equation (4)). This model uses no predictors and only a random intercept per subject in order to evaluate the proportion of variance in self-reported wellness that is accounted for by subject-specific intercepts. The result of ICC=0.563 and 95%CI=(0.371,0.671) suggests that 56.3% of the total variation in the data is accounted for by participant-specific differences. For example, some firefighters consistently rate themselves higher or lower than the population mean. The remaining 44% of variance reflects within-person fluctuations across training phases. These results motivate the approaches to personalization discussed in Section 6.3.2.

### 6.3. Predicting Perceived Wellness Ratings

Wellness rating prediction is treated as a regression problem where continuous values from 1 to 10 are later rounded to discrete classes. This is preferred over conventional classifiers, since the different classes are presumed to be *ordinal*, i.e., class 4 and 5 are closer together than 4 and 6. Note that the target variable has an imbalanced empirical distribution in this study (Figure 8a).

#### 6.3.1. Population-Level Model with Statistical Data Features

**Figure 11:**
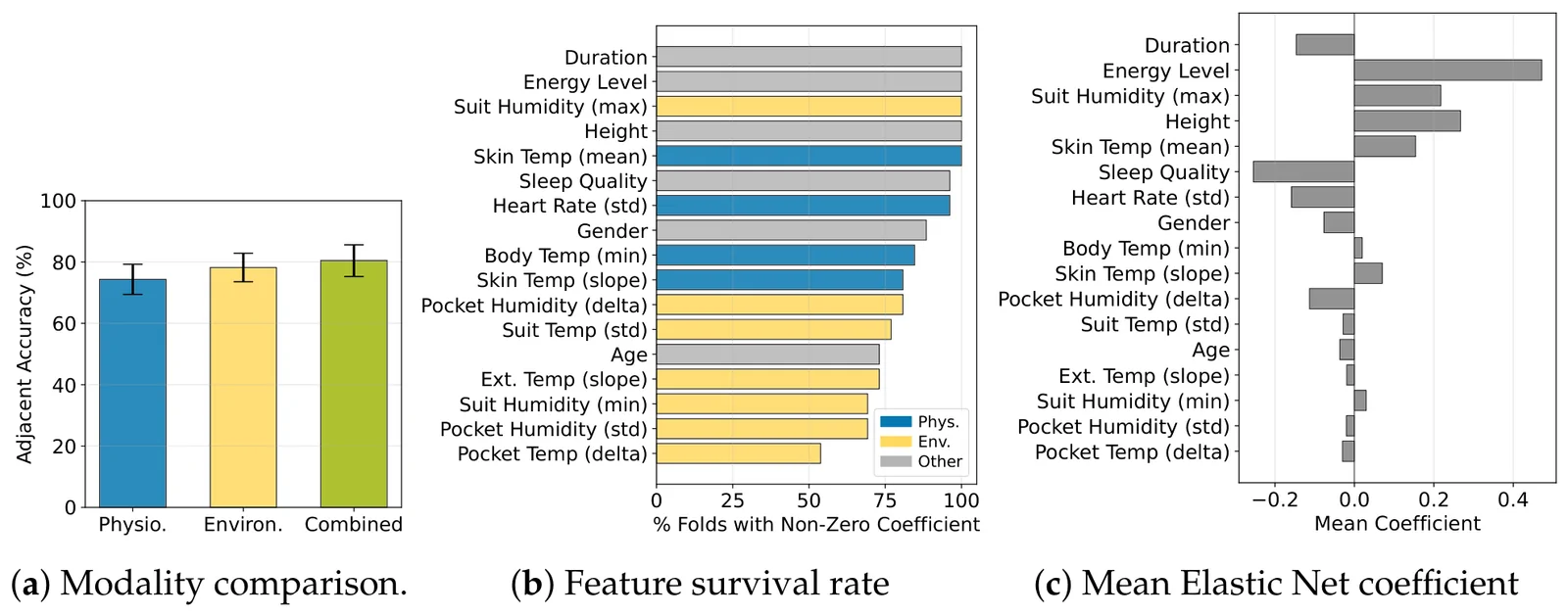
(**a**) Elastic Net model performance comparison as a function of data modalities included. (**b**) Percentage of LOSO folds in which each feature has a non-zero coefficient. Both physiological and environmental features survive L1 regularization alongside demographics. Colors indicate modality: physiological (blue), environmental (yellow), and demographic/other (gray). (**c**) Mean coefficient magnitude and direction across folds.

First, different configurations of an Elastic-Net model are tested, including models with access only to either physiological or environmental data features. The key results are presented in Table 7. For reference, Table 7 also includes a naive baseline that predicts the global mean wellness rating from the training data for every observation, which yields an MAE of 1.168. Similarly to the 4-class classifier (Section 6.1.3), the classifier which includes both modalities (“All Features”) performs better than the classifier with only environmental features, which still exceeds the performance of the model with only physiological features, when judged according to accuracy and adjacent accuracy. Figure 11a visualizes the performance. The model with access to all features has the highest adjacent accuracy, although it is not found to be a statistically significant difference in this dataset.

The table row labeled “Top Features” corresponds to the best-performing model after a grid search over the top *N* features to include (Section 5.2). The grid search operates in increments of 5 features from 5 through 65, where the “top” features are selected based on *survival rate* across LOSO folds. Each fold excludes one particular firefighter out of 26 from the training data and fits an EN model. Surviving features are those that have a non-zero coefficient. The survival rate counts the proportion of LOSO folds in which a particular feature survives. Although the individual EN fits exclude their held-out firefighter, the cross-fold ranking was determined after aggregating all LOSO fits. Therefore, note that every participant’s data contributed to the overall feature ranking. The top-10 model achieves an adjacent accuracy of 89.4%, exact accuracy of 45.5%, and MAE of 0.771. These values are interpreted as an exploratory comparison on this dataset because the cross-fold ranking and feature-set size were determined after aggregating the LOSO fits. The confusion matrix for this model is shown in Figure 6c. The selected features are as follows, grouped by type, with the rank in parentheses:

- Physiological: skin_temp_mean (4), hr_std (7), body_temp_min (9);
- Environmental: suit_humid_max (3), pocket_humidity_delta (10);
- Other: energy_level (1), height_inches (2), duration_sec (5), sleep_quality (6), gender (8).

Figure 11b,c illustrate the feature survival rates and the magnitudes of their coefficients, respectively.

The per-subject prediction errors are computed against the ground truth wellness ratings. To better understand the model failures, the Spearman rank correlations are computed between the residuals and the entire set of possible sensor and demographic data features. The top five highest correlated features are gender (ρ=0.244), suit humidity slope (ρ=0.236), height (ρ=−0.217), suit humidity standard deviation (ρ=0.202), and pocket humidity delta (ρ=−0.193). Out of these features, those that survived feature selection include gender, height, and pocket humidity delta. The fact that including these features in the model is not enough to resolve the errors motivates a mixed-effects modeling approach.

#### 6.3.2. Mixed-Effects Model for Participant-Specific Intercepts

While the population-level EN model shows preliminary success in predicting subjective wellness scores, earlier results suggest that subject-specific effects play an important role. Section 6.2 found that over 56% of the variance in wellness scores is accounted for by subject-specific differences. Therefore, a personalized modeling framework is necessary to account for this subjectivity. This section presents the results of the mixed-effects model and the subject-specific intercept estimation described in Section 5.3.

**Figure 12:**
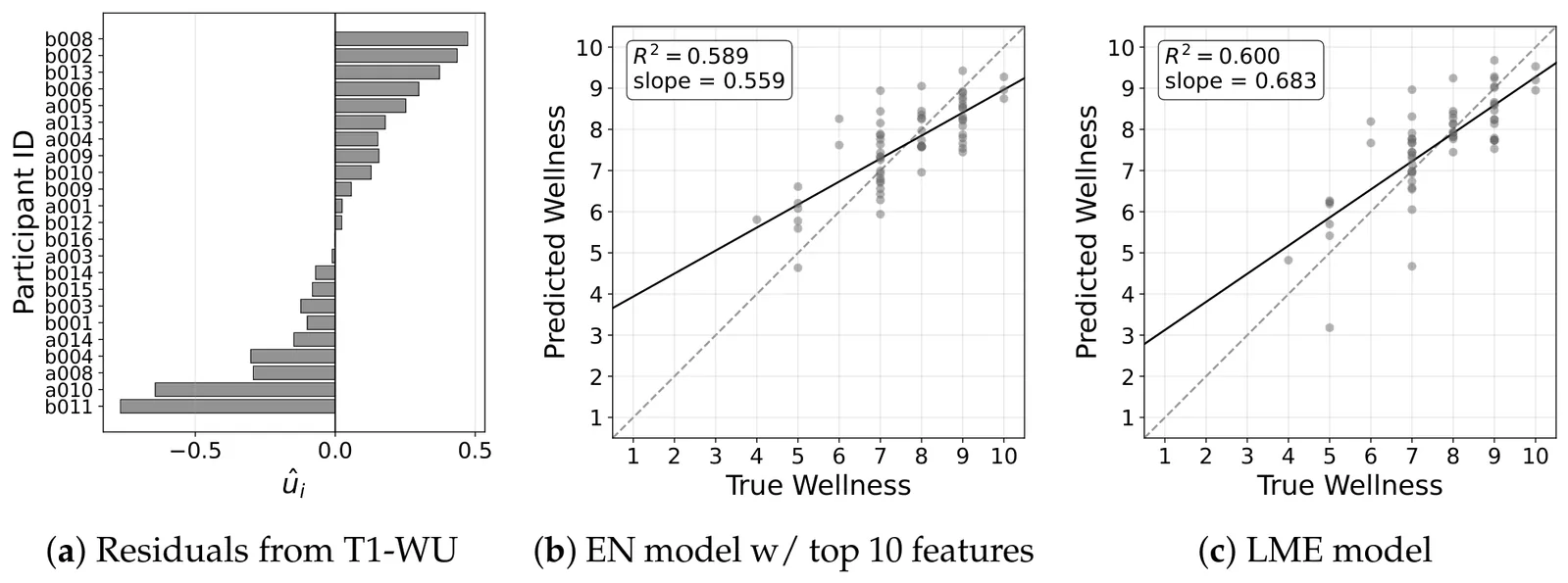
Mixed-effects model results.

First, Figure 12a shows the estimated person-specific random intercepts (${\hat{u}}_{i}$) derived from the T1-WU phase residuals (Equation (3)). These results reflect the large range of model prediction errors across the group of firefighters. The results before and after applying the intercepts to correct the residuals are shown in Figure 12b and Figure 12c, respectively. Both plots compare the true perceived wellness scores to those predicted by the model, including only the final three training phases after T1-WU. The perfect model would have slope equal to 1.0 and a coefficient of determination, $R^{2}$, equal to 1.0. The LME predictions align more closely with the ideal fit, improving the slope from 0.559 in the EN-model to 0.683 and the $R^{2}$ from 0.589 to 0.60. To isolate the effect of one-label personalization, the calibrated and uncalibrated LME predictions were evaluated on the same 61 later-phase observations from 23 held-out participants. Without access to a rating from the test participant, the LME fixed-effects predictions had an MAE of 0.732±0.327. After BLUP calibration using the participant’s first rating, MAE decreased to 0.658±0.371 (95% CI: 0.511–0.814), with an adjacent accuracy of 92.8±16.9% and exact accuracy of 53.6±36.4%. The participant-level mean reduction in MAE was 0.076 (95% CI: 0.023–0.132), and calibration reduced MAE for 17 of 23 participants (two-sided Wilcoxon signed-rank p=0.0075). A simple “first-label” baseline, which predicted every later rating as equal to the first rating, had an MAE of 1.029±0.775. These results indicate that the first rating provided useful participant-specific calibration, though this personalization relies on obtaining one ground-truth measurement.

For a paired comparison, both models were evaluated on the same 61 post-calibration observations from 23 participants. Mean MAE was 0.699 (95% CI: 0.581–0.822) for EN and 0.658 (95% CI: 0.511–0.817) for LME + BLUP. Across participants, MAE had a median of 0.685 (IQR: 0.450–0.940) for EN and 0.620 (IQR: 0.356–0.889) for LME + BLUP. The paired mean difference was 0.041 (95% CI: −0.039–0.110, where positive indicates a higher EN mean), and LME + BLUP had lower MAE for 14 of 23 participants. The modest average improvement suggests a potential benefit of using the first warm-up phase to estimate an offset in perceived wellness for each firefighter. However, the improvement from the EN-model to the LME model is not statistically significant in this study: a one-sided sign test on MAE gives p=0.2024, and a one-sided Wilcoxon signed-rank test on MAE gives p=0.0557.

## 7. Discussion

There are several important limitations of this study. Foremost is the small size of the experimental dataset, which includes 87 observations from 26 subjects collected during controlled training exercises at a single site. This limits the generalizability and statistical power of the findings, especially given the broad confidence intervals and variation in participant-level errors. The experimental design does not permit any analysis on the impact of training site, training duration, or number of training repetitions. This study also did not evaluate transfer across fire departments, equipment configurations, climates, or incident types. These differences in setting and population could lead to different outcomes and findings. It is not yet possible to generalize any results to real-world firefighting settings.

The finding that average perceived wellness did not decrease across successive training phases despite rising heart rate and body temperature may also be influenced by participants’ prior knowledge of the two-round protocol. The primary target is also a broad rating that may combine several factors. The self-reported wellness ratings in this study have an imbalanced empirical distribution concentrated in the upper portion of the 1–10 scale, which violates any assumption of normally distributed variables in significance tests. Regarding the analysis procedure, the present work selects a model using a top-10 feature set found by aggregating feature survival rates across all LOSO folds. For this model, the feature selection is not entirely independent of each held-out participant. It is meant to be a retrospective analysis of the current experimental data rather than a proposed training pipeline for a fully locked predictive model. The current results may therefore be optimistic for a fully blind setting. Future work with ample training data should pre-specify the feature set or use a feature-selection process that is fully blind to the held-out participant to evaluate the performance on an independent test cohort. Additionally, the calibration procedure proposed with the LME model requires observing one wellness rating from a new firefighter before personalized predictions can be made, which may not be practical in operational settings. Furthermore, because all observations were collected within a single session, the model assumes each firefighter’s intercept is stable over time. A longer-term study would be necessary to confirm if it remains consistent across days or weeks. Although the platform supports real-time sensor acquisition and visualization, real-time prediction and early warning were not evaluated because the models use summary features from complete activity phases. Models using causal sliding-window or partial-phase features would be required before the system can be used for real-time wellness prediction.

Future work should validate the system in uncontrolled operational fire environments across multiple sites, departments, equipment configurations, climates, participant populations, and incident or training scenarios. Additional studies should evaluate sliding-window or partial-phase prediction and explore online adaptation strategies that refine the subject-specific intercept as more observations become available. Although future studies may produce different findings, the proposed system retains value as a platform for collecting synchronized physiological and environmental measurements under demanding conditions.

## 8. Conclusions

This study presents a wireless multimodal sensing system worn by active firefighters that synchronizes physiological and environmental measurements. The system is validated on data from a new human-subjects experiment conducted with 28 firefighters during a live-fire training exercise. Models are trained from this data to classify warm-up phases versus live-fire phases with 97.7% accuracy and, furthermore, to predict which of the four phases a firefighter is in with 86.2% accuracy. The primary target variable in this study is the response to a survey question: “On a scale from 1–10, how well do you feel?” Statistical analyses present evidence that, in this experiment, self-reported wellness did not consistently track the measured physiological indicators. While heart rate and body temperature increased from Trial 1 to Trial 2, consistent with accumulated physiological strain, the self-reported wellness scores trended up on average over the course of the experiment. Several modeling approaches were compared for self-reported wellness prediction, including population-level elastic-net models and a naive global-mean baseline. In the exploratory comparison reported here, the one-label personalized LME model achieved an MAE of 0.658±0.371 on later-phase ratings, compared with 0.732±0.327 for the same model without personalization. This result suggests that one initial wellness rating can improve subsequent predictions by accounting for participant-specific reporting differences, although the method requires that rating before personalized prediction can begin. Overall, the multimodal sensing system shows promise as a research platform for firefighter monitoring and personalized self-reported wellness prediction. After further testing, the system could serve as a human-supervised decision-support tool, offering firefighters, incident commanders, and safety personnel data-driven monitoring to complement professional judgment and established safety procedures.

## Figures and Tables

**Table 1:** List of sensors with descriptions.

Classification	Sensor Info	Data Modalities	Description
Physiological	COROS Heart Rate Monitor	Heart rate	Optical heart rate monitor
Physiological	Greenteg CORE Sensor	Core body temp, Skin temp	Internal body and skin temperature
Environmental	ENS210 (Sensor Maestro SFM2)	Suit temp, Suit humidity	Internal suit temperature and relative humidity
Environmental	Adafruit 3245 (Omega)	External temp	K-type thermocouple, stainless steel sheath, external temperature
Environmental	SHT-30 (Adafruit Feather Sense)	Pocket temp, Pocket humidity	Board temperature and relative humidity, worn in outer pocket

Manufacturers and source locations: COROS Wearables, Inc. (Irvine, CA, USA); greenteg AG (Zurich, Switzerland); SensorMaestros (Denver, CO, USA); Adafruit (Brooklyn, NY, USA); Omega Engineering (Norwalk, CT, USA).

**Table 2:** Binary classification of training phase (WU or LF) using different feature sets.

Feature Set	# Features	Accuracy	F1	Precision	Recall	AUPRC
All Sensors	56	97.7%	0.977	0.978	0.978	0.997
Environmental Only	35	97.7%	0.977	0.978	0.978	0.996
Physiological Only	21	82.8%	0.828	0.857	0.800	0.866

**Table 3:** Wilcoxon signed-rank tests of sensor data mean-value features and wellness target variable between live-fire and warm-up phases.

Variable	Live Fire (Mean)	Warm-Up (Mean)	Diff	*p*-Value
Wellness	7.96	6.91	1.04	0.0001 *
Skin Temp	36.01	33.52	2.49	0.0000 *
Suit Temp	34.80	30.91	3.90	0.0000 *
Suit Humidity	74.66	63.60	11.07	0.0000 *
Ext. Temp	42.67	17.77	24.89	0.0000 *
Pocket Humidity	42.35	35.82	6.53	0.0000 *
Pocket Temp	24.02	22.77	1.25	0.0004 *
Heart Rate	147.44	156.42	−8.97	0.0013 *
Body Temp	37.91	37.78	0.13	0.0091 *

* Indicates p<0.05.

**Table 4:** Four-class classification of training phase type and trial number using different feature sets.

Feature Set	# Features	Accuracy	F1-Macro	Precision	Recall
All Sensors	56	82.8%	0.829	0.830	0.829
Environmental Only	35	74.7%	0.748	0.748	0.750
Physiological Only	21	64.4%	0.643	0.647	0.643

**Table 5:** Comparison between Trial 1 Warm-Up and Trial 2 Warm-Up (Wilcoxon signed-rank).

Variable	T1-WU (Mean)	T2-WU (Mean)	Diff	W	*p*-Value	r
Wellness	6.67	6.89	0.22	25	0.4487	0.242
HR mean (bpm)	151.87	160.04	8.17	28	0.0202 *	0.634
HR std (bpm)	14.97	12.37	−2.60	22	0.0079 *	0.712
Body temp mean (C)	37.34	38.11	0.77	8	0.0004 *	0.895
Body temp std (C)	0.08	0.09	0.01	66	0.6441	0.137

* Indicates p<0.05.

**Table 6:** Linear mixed-effects model incorporating demographics variables.

Fixed Effect	Coef.	SE	z	*p*-Value	95% CI
Intercept	7.399	0.163	45.53	0.0000 *	(7.081, 7.718)
Age	−1.206	0.423	−2.85	0.0044 *	(−2.036, −0.377)
Experience	0.951	0.435	2.19	0.0287 *	(0.099, 1.803)
Height	0.775	0.194	4.00	0.0001 *	(0.395, 1.156)
Weight	−0.036	0.173	−0.21	0.8327	(−0.375, 0.302)

* Indicates p<0.05.

**Table 7:** Overall results comparison between different approaches on the target variable prediction task: “How well do you feel? (scale 1–10).” Metrics are reported as mean ± standard deviation across LOSO folds. The best results in each experiment group are in bold.

Model	Configuration	# Features	Adj. Acc. (%)	Acc. (%)	MAE
Elastic Net Modality Fusion	Physio Features	30	74.4 ± 25.1	24.0 ± 28.1	1.099 ± 0.447
	Env Features	44	78.2 ± 23.7	26.9 ± 25.9	0.963 ± 0.443
	All Features	65	80.4 ± 26.3	27.2 ± 25.9	1.013 ± 0.474
	Top Features	10	**89.4 ± 15.8**	**45.5 ± 30.9**	**0.771 ± 0.312**
Mixed-Effects	LME (Zero-Label)	10	89.9 ± 18.2	37.0 ± 31.1	0.732 ± 0.327
	LME + BLUP (One-Label)	10	**92.8 ± 16.9**	**53.6 ± 36.4**	**0.658 ± 0.371**
Baseline	First-Label		79.0 ± 32.7	29.7 ± 28.6	1.029 ± 0.775
Baseline	Naive (Global Mean)		62.1	29.9	1.168

## Data Availability

The data presented in this study are available on request from the corresponding author due to privacy restrictions.

## Abbreviations

The following abbreviations are used in this manuscript:

AUPRC	Area Under the Precision-Recall Curve
BLE	Bluetooth Low Energy
BLUP	Best Linear Unbiased Prediction
ECG	Echocardiogram
EN	Elastic Net
HR	Heart Rate
HRV	Heart Rate Variability
ICC	Intraclass Correlation Coefficient
LF	Live Fire
LME	Linear Mixed-Effects
LOSO	Leave-One-Subject-Out
LR	Logistic Regression
MAE	Mean Absolute Error
MLP	Multilayer Perceptron
MSE	Mean Squared Error
PPE	Personal Protective Equipment
REML	Restricted Maximum Likelihood
RPE	Rating of Perceived Exertion
SOTA	State-Of-The-Art
T1	Trial 1
T2	Trial 2
WU	Warm-Up
